# Effects of Dried Distillers Grains in Supplements for Beef Cows During Late Gestation on Cow–Calf Performance and Metabolic Status

**DOI:** 10.3390/ani15121698

**Published:** 2025-06-08

**Authors:** Johnnatan Castro Cabral Gonçalves, Jean Marcelo Albuquerque, Edinael Rodrigues de Almeida, Luanna Carla Coelho, José Augusto Moura Godinho, Lilian Yukie Pacheco Toma, Matheus Fellipe de Lana Ferreira, Luciana Navajas Rennó, Cláudia Batista Sampaio, Edenio Detmann, Sidnei Antônio Lopes

**Affiliations:** 1 Departament of Animal Science, Federal University of Viçosa, Viçosa 36570-900, MG, Brazil; jean.albuquerque@ufv.br (J.M.A.); luannacoelhozoo@gmail.com (L.C.C.); jose.godinho@ufv.br (J.A.M.G.); lilian.toma@ufv.br (L.Y.P.T.); lucianarenno@ufv.br (L.N.R.); claudiabsampaio@ufv.br (C.B.S.); detmann@ufv.br (E.D.); sidnei.lopes@ufv.br (S.A.L.); 2 Department of Animal Science, Southwest Bahia State University, Itapetinga 45700-000, BA, Brazil; edinael.almeida@ufv.br; 3 Hill Farm Research Station, Louisiana State University, Homer, LA 71040, USA; mferreira@agcenter.lsu.edu

**Keywords:** metabolizable protein, ethanol byproduct, negative energy balance, rumen undegradable protein, fetal programming

## Abstract

Forty multiparous Nellore cows, with an average body weight, body condition score, and gestation days of 533 ± 32 kg, 5.7 ± 0.4 points, and 198 days, respectively, were managed under continuous grazing on a *Urochloa decumbens* pasture and either received or did not receive 1 kg/day of supplement varying in the levels of inclusion of dried distillers grains (DDG) (Control, 0% DDG, 42% DDG, or 84% DDG) during the last trimester of gestation. The objective was to evaluate the effects of prepartum DDG supplementation on performance, reproduction, fetal development, and calf performance. A quadratic effect of supplementation on BW variation pre- and postpartum was observed (*p* < 0.05), with cows treated with 42% DDG showing higher BW gain prepartum and low BW loss postpartum. Cows supplemented with 42% DDG had lower levels of non-esterified fatty acids and β-hydroxybutyrate prepartum (*p* < 0.05). However, body weight-related metrics, such as condition score, carcass traits, and metabolic indicators, varied only by measurement day (*p* < 0.05). No effect of supplementation was observed on fetal development, calf metabolic profile, milk yield, or postnatal calf performance (*p* > 0.05). It is recommended to include up to 42% DDG in supplements for beef cows during late gestation grazing on low-quality tropical forage conditions.

## Simple Summary

Supplementing beef cows in tropical pastures with dried distillers grains (DDG) during the late gestation period provides significant benefits. In our study, Nellore cows that received 1 kg of supplement containing 42% of DDG showed greater weight gain prepartum, less weight loss postpartum, and better metabolic indicators related to nutritional status prepartum. The supplementation did not affect reproduction, fetal development, milk production, or calf growth. These results can help producers evaluate the technical viability of using DDG for beef cows in tropical systems. Additionally, they contribute to advancing scientific knowledge on ruminant nutrition in tropical conditions.

## 1. Introduction

The negative energy balance is critical during the peripartum period, as homeorhetic adaptations are necessary to support fetal development and initial milk production [1,2,3]. This condition can be exacerbated in tropical beef cattle production due to the reduction in voluntary intake near calving and the nutritional constraints associated with the seasonal variation in forage quality.

Improving the nutritional status of beef cows during the prepartum period to ensure an adequate body condition score (BCS) at calving can benefit cow–calf systems by reducing the calving interval, shortening the postpartum anestrus period, enhancing conception rates, and promoting fetal development [4,5,6,7]. Under tropical conditions, strategic supplementation programs can be effectively implemented during the last trimester of gestation to ensure an adequate BCS at calving, even when forage resources are of low nutritional value. This approach is viable because the final trimester of gestation typically coincides with weaning, a phase when maternal nutrient requirements are relatively lower due to the reduction in lactation demands [8].

The inclusion of coproducts from the sucroenergetic industry in animal nutrition can be considered sustainable from social, environmental, and economic perspectives [9]. Among these coproducts, dried distillers grains (DDG) are particularly valuable for their nutritional profile, characterized by a high concentration of rumen undegradable protein (RUP) and digestible fiber and a low fat content [10]. Thus, they can be a direct source of metabolizable protein and fermentable compounds. However, the primary demand of rumen microorganisms for nitrogen compounds raises questions about the efficiency of RUP in supplements designed for beef cattle grazing on low-quality forages. Improvements in the utilization of low-quality fibrous feeds can be achieved through protein supplementation [11,12], resulting in a greater weight gain and body condition score in cows [13]. A certain amount of latent energy can be extracted from low-quality fibrous compounds when the nitrogen requirements of rumen microorganisms are met. On the other hand, when the ruminal nitrogen demand is not met, recycling events are exacerbated, which can reduce the efficiency of metabolizable protein utilization.

The effects of RUP supplementation combined with non-starch carbohydrates under low-quality forage conditions remain poorly understood, particularly for cows in the last trimester of gestation. For overfed beef cows, the potential benefits of such supplementation include improved prepartum performance, enhanced protein metabolic status, and increased adipogenic and myogenic potential in offspring [14]. For steers grazing low-quality pasture, the intake of distillers grains improved ruminal metabolism [15], while for cows in a nutritionally restricted state, the supplementation of distillers grains may improve performance and body condition from calving to the breeding season [16]. Additionally, when economically feasible, replacing conventional true protein sources with DDG and adjusting the supply of rumen degradable protein (RDP) with urea can be an effective strategy for finishing systems under conditions of low-quality forage availability [17].

We hypothesize that the inclusion of DDG in supplements offered during late gestation would lead to improvements in the metabolic status and productive performance of beef cows during the peripartum period, due to its higher RUP content and potential to increase the levels of metabolizable protein in the diet. Furthermore, this nutritional modulation could also affect the performance and metabolism of offspring. The objective of this study was to evaluate the effects of DDG in supplements during late gestation on the performance, reproductive, and metabolic parameters of beef cows during the peripartum period and their progeny.

## 2. Materials and Methods

This experiment was conducted between June and November 2022 at the Beef Cattle Teaching, Research, and Extension Unit of the Federal University of Viçosa, Minas Gerais, Brazil. All procedures involving animals were previously approved by the Animal Use Ethics Committee of the Federal University of Viçosa (protocol number 11/2022).

### 2.1. Animals, Experimental Design, and Treatments

Forty multiparous Nellore cows at 198 days of gestation (inseminated on the same day), parity order of 4 ± 2, with a body weight (BW) of 533 ± 32 kg and BCS 5.7 ± 0.4, were randomly allocated into eight pastures of *Urochloa decumbens* (2.7 ha and five cows/pasture), previously deferred. The cows were managed in paddocks under a continuous grazing system, with a fixed stocking rate of 2.2 animal units per hectare. Pastures were equipped with covered feeders and water troughs.

Each group of five animals was formed, and treatments were randomly assigned to the groups and consisted of the following: Control—animals receiving mineral mixture *ad libitum*; and supplemented—those with supplements containing different levels of DDG, 0% DDG, 42% DDG, or 84% DDG (Table 1). The supplements were formulated to contain 40% crude protein (CP) on a dry matter (DM) basis and were offered daily at a rate of 1 kg/animal to meet about 35% of the requirements for CP [8]. The supplement was provided for 85 days on average prior to calving, always at 11:00 a.m. After calving, the groups were maintained and received mineral mixture *ad libitum*. Postpartum data collection occurred over 60 days, considering the specific calving date of each animal.

### 2.2. Productive Performance

The cows were weighed on d-90, d-60, d-30, d-1, d1, d30, and d60 relative to calving (d0). The calves were weighed on days d1 and d60 after birth. On d-90, d1, and d60, the BCS, on a scale of 1 to 9 [18], was assessed by five trained evaluators, and carcass ultrasonography was performed. Images of the *Longissimus dorsi*, taken between the 12th and 13th ribs and between *Gluteus medium* and *Biceps femoris*, were captured using an ultrasound device (SSD 500V®, Aloka, Ltd., Tokyo, Japan ) equipped with an 18 cm linear probe. The ribeye area (RBA) and subcutaneous fat thickness at the rump (RFT) were measured using the BioSoft Toolbox® II software for Beef (Biotronics Inc., Ames, IA, USA).

The relative variations in the productive performance measures of the cows (BW, BCS, RBA, and RFT) were calculated for the prepartum and postpartum periods, considering the initial measurements (d-90) and those at calving (d-1) and from calving (d-1) to the final measurement (d60), respectively. The following equations are the formulas used for calculation:

$$BW,BCS,RBA\;or\;RFT\;prepartum\;variation=\frac{Calving-Initial}{Initial}$$

$$BW,BCS,RBA\;or\;RFT\;postpartum\;variation=\frac{Final-Calving}{Calving}$$

where BW, BCS, RBA, or RFT prepartum/postpartum variation indicates the variable analyzed, in percentage (%); calving, initial, and final indicate the moment of the measurement, corresponding to d-1, d-90, and d60 relative to calving, respectively.

To estimate milk production, cows were milked in the 3rd and 5th weeks after calving. Milking procedures were performed with a controlled suckling period prior to calf separation. Cows were milked at 5:00 a.m. after being separated from their calves for twelve hours, followed by the administration of 1 mL of oxytocin (10 IU/mL, Ocitopec^®^, Biovet, São Paulo, Brazil). Daily milk yield and the above-mentioned procedures were based on the morning milk yield adjusted to 24 h according to Lopes et al. [19].

### 2.3. Estimation of Forage and Supplement Availability and Quality

Every 14 and 28 days, forage samples were collected to evaluate the quality and forage mass availability, using the hand-plucking technique and the square technique, respectively. The samples for forage mass were collected by cutting at ground level in four representative areas of each paddock, delimited to 0.25 m^2^, to quantify DM allowance and potential digestible dry matter (pdDM) allowance. The samples were weighed, oven-dried (55 °C), and then processed in Willey-type mills, using 1 mm and 2 mm screens. The pdDM was estimated according to the following equation:

pdDM=0.98×(100−NDF)+(NDF−iNDF)

where pdDM is the potentially digestible dry matter content (as % of DM), 0.98 is the true digestibility coefficient of the cell contents, NDF and iNDF are the forage contents of neutral detergent fiber and indigestible neutral detergent fiber, respectively (as % of DM).

Forage and supplement samples were analyzed according to the standard analytical procedures of the Instituto Nacional de Ciência e Tecnologia—Ciência animal (INCT, CA) [20]. The following were evaluated: dry matter content (DM, INCT-CA method G-003/1), mineral matter (MM, INCT-CA method M-001/2), CP (CP, INCT-CA method N-001/2), neutral detergent fiber corrected for ash and protein (apNDF, INCT-CA method F-013/1), neutral detergent insoluble protein (INCT-CA method N-004/2), neutral detergent insoluble ash (INCT-CA method M-002/2), and indigestible neutral detergent fiber, determined by in situ incubation in non-woven fabric bags (100 g/m^2^) for 288 h, with samples processed through a 2 mm sieve (iNDF, INCT-CA method F-009/2). The RDP and RUP concentrations of the supplements were estimated based on the tabulated values in Valadares Filho et al. [21].

### 2.4. Blood and Skeletal Muscle Collection: Processing and Analysis

Blood samples were collected from the jugular vein, prior to supplementation, in vacuum tubes with a separator gel and clot accelerator, on d-30, d-15, d1, d15, d30, d45, d51, and d60. Calf blood was collected on d1 and d15 relative to birth. After collection, the samples were centrifuged at 2500 × *g* for 15 min to obtain blood serum. Serum was frozen at −20 °C in microtubes for subsequent analyses. Cow and calf sera were analyzed for the concentrations of total protein (K031-Biuret Method, Bioclin, Belo Horizonte, Brazil), albumin (K040-Bromocresol Green Method, Bioclin), and glucose (K082-Enzymatic Colorimetric Method, Bioclin). Additionally, cows’ serum was analyzed for urea (K056-UV kinetic method, Bioclin) and total cholesterol (K083-Enzymatic Colorimetric Method, Bioclin). Non-esterified fatty acids (NEFAs) and β-hydroxybutyrate (βHB) were evaluated in cows’ serum using Randox kits, Antrim, UK (FA115-Colorimetric Method and RB1007-Enzymatic Method, respectively). All above-mentioned analyses were performed at the Animal Physiology Laboratory of the Department of Animal Science at the Federal University of Viçosa using an automated biochemical analyzer (Mindray, BS200E, Shenzhen, China). Insulin-like growth factor type 1 (IGF-1) was quantified on d-30, d-15, d1, d15, d30, and d51 for cows and on d1 for calves using DiaSorin kits, California, USA, in an automated chemiluminescence analyzer (Liaison XL, Saluggia, Italy).. Cows’ and calves’ globulin concentrations were calculated using the difference between total protein and albumin. Serum urea nitrogen (SUN) was considered to make up 46.67% of serum urea.

*Longissimus dorsi* muscle samples, located between the 10th and 11th ribs, were collected by biopsy from the offspring at 55 days of age. This procedure was carried out with the appropriate veterinary care, including the application of local anesthesia (Lidocaine 2%, LidoVet, Bravet, Rio de Janeiro, Brazil) and suturing after collection. The animals were monitored and treated with antibiotics and anti-inflammatory drugs, and the sutures were removed two weeks later. Approximately 1 cm^3^ of muscle was collected and fixed in 10% formalin for histological analysis. The samples were dehydrated using a growing ethanol series (70% to 100%), followed by embedding in xylene and, subsequently, in liquid paraffin for block preparation. Sections of 5 μm were obtained using a rotary microtome and stained with hematoxylin–eosin. The slides were mounted in Entellan (Merck, Darmstadt, Germany) and prepared for analysis. To observe muscle cell area, digital images of the muscle sections per animal were obtained using an EvosFL microscope (ThermoScientific, Waltham, USA)and analyzed using ImageJ, 1.54v software (National Institute of Health, Baltimore, MD, USA). To obtain the average muscle fiber area for each sample, measurements were made in 30 different cells in different regions of the slide containing the muscle tissue sample.

### 2.5. Measures and Breeding Season Protocol

The breeding season started at 61 ± 5 days after calving and involved two artificial inseminations. Cows were synchronized using the following protocol: On day −10 relative to insemination, a slow-release progesterone intravaginal device (Primer, Tecnopec, São Paulo, Brazil) was inserted, and an intramuscular injection of 2.0 mg of estradiol benzoate (RIC-BE, Tecnopec) was administered. On day −2, the intravaginal device was removed, and the cows received an intramuscular injection of 0.48 mg of sodium cloprostenol (Estron, Tecnopec), 300 IU of equine chorionic gonadotropin (Novormon, Zoetis-Pfizer, Campinas, Brazil), and 1 mg of estradiol cypionate (E.C.P., Zoetis-Pfizer). Timed artificial insemination (TAI) was performed 46–52 h after the removal of the intravaginal device on the insemination day (D0). Semen from five Nellore bulls was randomly assigned to each cow. The protocol was repeated 14 days after the insemination (D14) in the same manner. On day +22, the implant was removed, and pregnancy diagnosis was performed by color Doppler ultrasound (Mindray, DP 50). Non-pregnant cows received an intramuscular injection of 0.48 mg of sodium cloprostenol (Estron, Tecnopec), 300 IU of equine chorionic gonadotropin (Novormon, Zoetis-Pfizer), and 1 mg of estradiol cypionate (E.C.P., Zoetis-Pfizer). TAI was performed again 46–52 h after the removal of the intravaginal device on day 24. A new pregnancy diagnosis was conducted 30 days later, confirming pregnancies from both the first and second inseminations. The pre-ovulatory follicle diameter (POFD) at the first insemination was measured as reproductive success.

### 2.6. Statistical Analysis

Response variables related to productive performance and the metabolic profile of cows over time were analyzed using the GLIMMIX procedure in SAS 9.4 (Inst. Inc., Cary, NC, USA). The MIXED procedure of SAS was used to analyze response variables related to the calves and variations in productive performance measures (BW, BCS, RBA, and RFT variation) for milk production and POFD.

The models included the fixed effects of treatment, day, and their respective interactions. For the variables measured in the calves, the effect of sex was controlled. The effect of the group nested within each treatment was considered a random effect. A general model for the analyzed variables in cows can be represented by the following equation:

Yijk=µ+Ti+G(i)j+e(ij)k

where Yijk = response measured in animal “k”, belonging to the experimental unit (group) “j” and subjected to treatment “i”; µ = overall constant; Ti = effect of treatment “i”; G(i)j = effect of group “j” nested within treatment “i”; e(ij)k = unobserved random error, assumed to be NID (0; σ^2^).

For the variables analyzed in calves, the model included the effect of sex. For all variables, means were compared with adjustments for multiple comparisons, and contrasts were applied to evaluate specific comparisons between treatments and interactions, including the supplementation effect (Sup) and linear (L) and quadratic (Q) trends among treatments. Significance was declared at *p* < 0.05.

## 3. Results

The forage DM allowance during the pre- and postpartum periods was 71.6 and 67.6 g/kg of BW, respectively, corresponding to an average pdDM allowance of 44.6 g/kg BW. During these periods, the CP content of the pasture was 40.2 and 80.8 g/kg DM, while the average concentration of apNDF in the forage was 743 and 673 g/kg DM (Table 2).

**Figure 1:**
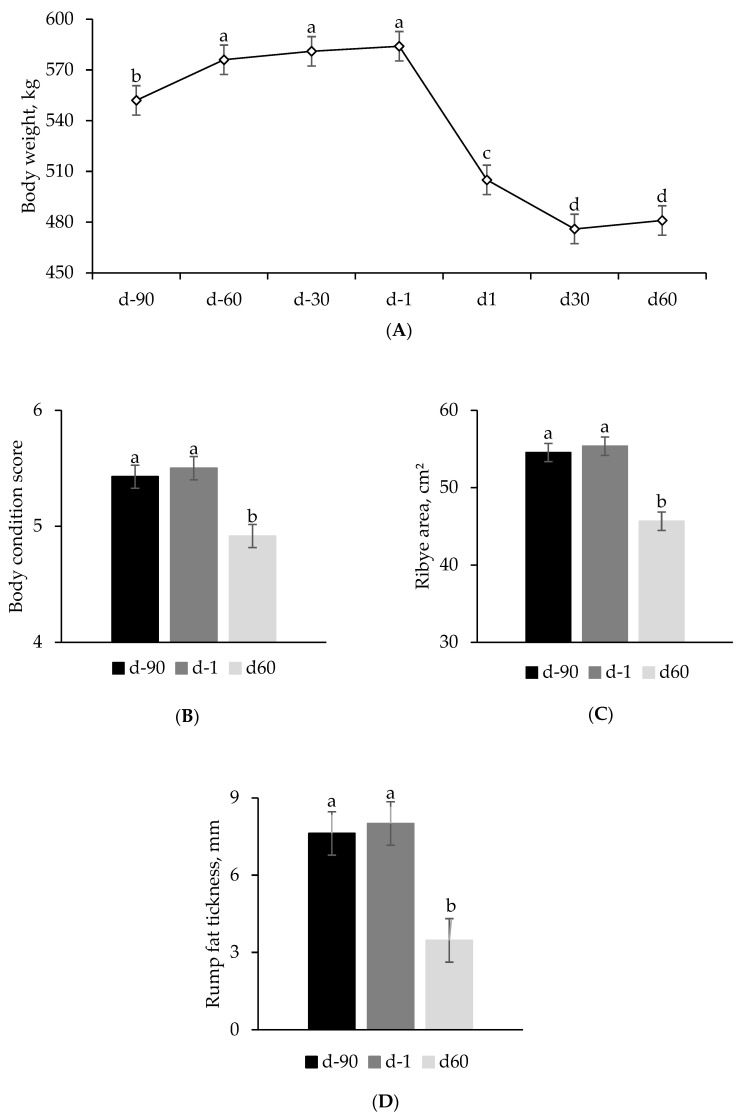
Body weight (**A**), body condition score (**B**), ribeye area (**C**), and rump fat thickness (**D**) measured throughout the peripartum period of Nellore cows supplemented or not with different levels of dried distillers grains during the last trimester of gestation. There was no treatment effect (*p* > 0.05) and no interaction between treatment and time (*p* > 0.05). ^a, b, c, d^ Repeated measures over time followed by different letters differ from each other with *p* < 0.05.

A quadratic effect (*p* = 0.047; Table 3) indicated a greater relative gain for the 42%DDG treatment compared to the Control and 84%DDG during the prepartum period. During the postpartum period, cows in the 42%DDG treatment lost less weight compared to the 0% DDG cows. Nevertheless, no other differences between treatments were found (*p* > 0.05) for variables related to productive performance, BCS, BCS change, RBA, RFT, or BW measured throughout the experiment (overall means shown in Figure 1). Additionally, reproduction was not affected by treatment. On average, the POFD was 16.3 mm, 16.7 mm, 16.6 mm, and 15.4 mm for the Control, 0% DDG, 42% DDG, and 84% DDG, respectively (*p* = 0.68). The average conception rate at the first artificial insemination was 79.9%, and only one cow from the 84%DDG group failed to conceive throughout the entire breeding season. 

A treatment × period interaction was observed for total cholesterol (p = 0.036), SUN (*p* < 0.001) βHB (*p* = 0.033), and NEFA (*p* = 0.012; Table 4). Total cholesterol concentration was higher for the 84% DDG treatment compared to the 0% DDG treatment on d1, while on d30 it was lower compared to the Control. Serum urea nitrogen concentration was higher in supplemented animals (*p* = 0.020), and there was a linear decrease with increasing DDG inclusion in the supplements (*p* = 0.005). Overall, during the prepartum period, SUN levels were higher for the 0% DDG group compared to the Control on d-30 and higher than those of all other treatments on d-15. In the postpartum period, cows fed 42% DDG had higher SUN levels than those in the Control and 84% DDG groups on d15. However, on d30, cows in the 0% DDG group had higher levels compared to those in the 42% DDG and 84% DDG treatments.

β-hydroxybutyrate levels did not differ between treatments during the postpartum period, however, during the prepartum period, the concentration of this ketone body was lower in the 42% DDG treatment compared to the 84% DDG treatment on d-30 and lower than that of the Control on d-15. Non-esterified fatty acids levels were generally lower in the 0% DDG and 42% DDG treatments on d-30 and lower for 0% DDG and 42% DDG compared to the Control on d-15. Lastly, on d1, cows in the Control group had higher NEFA levels than those in the 84% DDG treatment.

**Figure 2:**
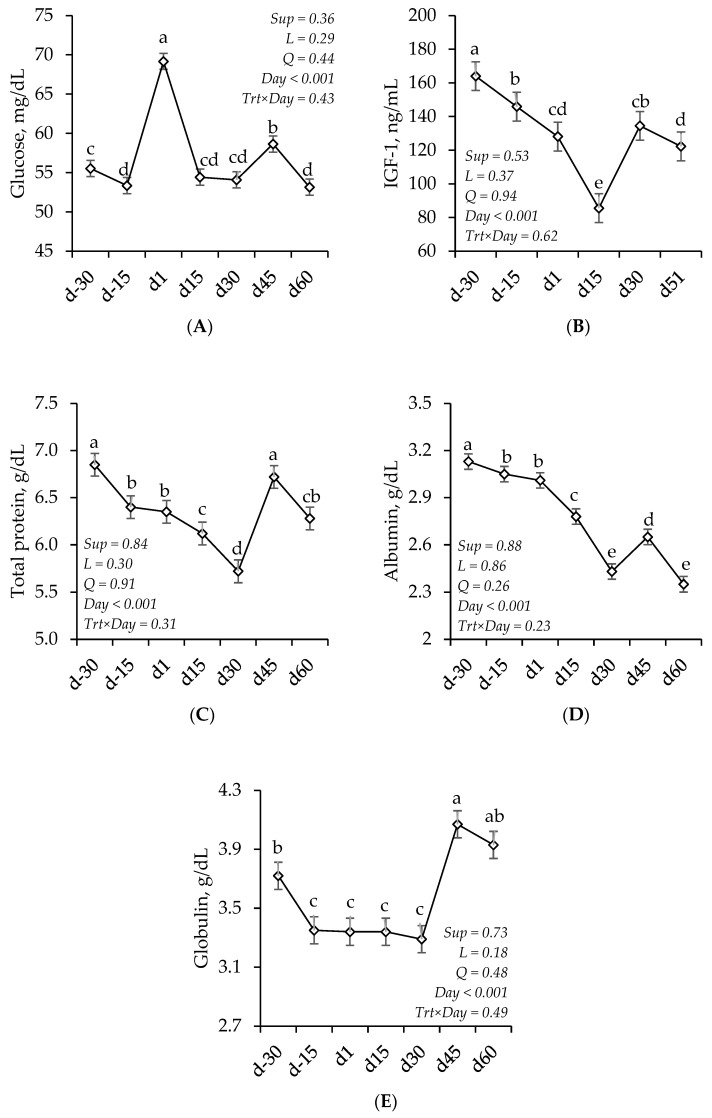
The concentrations of glucose (**A**), insulin-like growth factor 1 (IGF-1) (**B**), total proteins (**C**), albumin (**D**), and globulin (**E**) in Nellore cows supplemented or not during the final third of gestation with different levels of dried distillers grains. There was no treatment effect (*p* > 0.05) and no interaction between treatment and measurement day (*p* > 0.05). ^a, b, c, d, e^ Repeated measures over time followed by different letters differ from each other with *p* < 0.05.

The concentrations of IGF-1, glucose, total proteins, albumin, and globulin were not affected by treatment (*p* > 0.05) but varied according to collection day (*p* < 0.001; Figure 2). In general, glucose levels were the highest on d1, followed by d45, and were the lowest on d60. IGF-1 levels decreased from d-30 to d15, then increased from d15 to d30. Total protein and albumin concentrations decreased from d-30 to d30, then increased from d30 to d45. Globulins decreased from d-30 to d-15 and remained at a constant level until d30, when they increased between d30 and d45.

The cows gave birth to 27 male and 17 female calves. Two cows had twin male births, and one cow aborted early in the experiment, thus being removed from the experiment. The effect of sex was controlled in the variables measured in the calves and did not interfere with the results. Calves’ measurements and the milk yield of the dams are described in Table 5. Calves’ birth weight and the longitudinal section area of the *Longissimus dorsi* muscle were not affected by dams’ treatment (*p* > 0.05). No differences were found among maternal treatments for any of the serum biomarkers measured in the calves (*p* > 0.05). Maternal supplementation during the prepartum period also had no effect on postpartum milk yield (*p* = 0.33). Thus, differences were observed only for the day of sampling regarding the calf BW and serum profiles of total protein, albumin, and globulins (*p* < 0.01). Glucose levels were not affected by sampling day (*p* = 0.38), remaining similar on d1 and d15. The IGF-1 concentration measured one day after birth was also not influenced by treatment (*p* = 0.32). Overall, total protein and globulin levels were higher on d15 compared to d1, while albumin levels increased over this period. The average daily gain of calves from birth to 60 days of age was 0.79 kg.

## 4. Discussion

Forage availability did not represent a productive constraint, as it remained within the levels ranging from 40 to 60 g pdDM/ kg BW. Moreover, the observed levels were close to the forage allowance considered optimal by Sousa et al. [22], at 70.6 g DM/kg BW, to ensure the productive performance of beef cows during the peripartum period. However, the qualitative limitations of the forage, evidenced by low CP concentrations and the progressive increase in the levels of iNDF, especially up to September, when the calving peak occurred, suggest the potential occurrence of multiple nutritional deficiencies during this period, indicating that the forage was of low quality.

Although supplementation may alleviate negative energy balance and reduce the mobilization of reserves to meet the gestational component at late gestation, we did not observe differences between treatments in body condition measures assessed by the BCS and carcass ultrasound. Studies under similar conditions found no impact of low-intake supplement on performance [23,24,25]. However, this strategy may be effective for smaller-framed cows and those with a lower condition score, typically classified as a BCS below 5, due to their lower nutritional requirements [8,26].

Although we did not observe an overall effect of supplementation on body conditions, we detected a quadratic effect on BW variation. Cows supplemented with 42% DDG showed higher relative weight gain during the final third of gestation, while those supplemented with 84% DDG had lower gains, similar to the Control group. This effect was supported by the reduced levels of NEFA and βHB in prepartum cows supplemented with 42% DDG, indicating less mobilization of body reserves. Since these metabolites are directly associated with energy deficit and the mobilization of reserves [27], our results suggest that not supplementing and supplementing with levels above 42% DDG in low-consumption supplements are not effective in alleviating the negative energy balance in pregnant beef cows under tropical conditions, which is necessary to maintain the gestational component during the final third of gestation.

Both the availability of body reserves and nutritional intake during the final third of gestation are crucial for fetal development [4,5,7]. During this period, cows adjust their nutritional requirements to allocate more nutrients to the gestational component [2,8,28]. When cows have adequate body reserves, they can compensate for the adverse effects of mild nutrient deficiencies, resulting in the loss of maternal tissue. Thus, it is likely that the cows receiving 42% DDG allocated a greater amount of nutrients to the gestational component. However, the cows were generally in good body condition, and therefore, the controlled mobilization of their reserves was sufficient to mitigate the potential negative effects of maternal nutritional restriction on the fetus. This was evidenced by the similar birth weight and muscle fiber size of the offspring.

In low-quality pastures, a minimum amount of ruminal ammonia nitrogen is necessary to ensure optimal energy extraction from low-quality fibrous components by fibrolytic microorganisms. Similarly, nitrogen supplementation, combined with rapidly fermentable carbohydrates, also promotes the availability of metabolizable compounds such as volatile fatty acids and microbial protein. Corn processing increases the levels of RUP, while most non-fibrous carbohydrates are fermented to ethanol, concentrating the fibrous fraction in DDG [10]. Although this fraction is degradable, its degradation rate is slower compared to soluble carbohydrates. In our study, DDG supplements showed a gradual increase in fiber and indigestible fiber levels, along with a decrease in RDP. This behavior is associated with lower nitrogen availability in the rumen, which was reflected in the lower NUS concentrations between supplemented cows.

Thus, initially, the elevated levels of RUP, and consequently the lower nitrogen availability in the ruminal environment, may have compromised forage intake in cows from the 84% DDG treatment. This possibly resulted in the worst productive performance and serum energy metabolism indicators, similar to those observed in the Control during the prepartum period. On the other hand, when DDG was included at an intermediate dose (42%), the higher nitrogen availability in the rumen, combined with the presence of rapidly fermentable carbohydrates, may have favored energy extraction from fiber and microbial protein synthesis and, in addition to the RUP levels, increased the availability of metabolizable protein and energy for the cows. Although studies on ruminal metabolism in pregnant cows grazing low-quality pastures are scarce, this is supported by Picanço et al. [29], which indicate that DDG inclusion levels above 20% in supplements result in decreased forage intake, dry matter digestibility, and fibrous fraction digestibility. In cattle on essentially forage-based diets, this reduction can impact productive performance. Furthermore, when ruminal requirements are met, the increase in RUP can positively modulate nutrient delivery to the fetus and improve weight gain in pregnant cows.

Overall, the animals experienced postpartum body condition loss, with continuous weight loss observed until d30, a period in which intake capacity falls short of its potential while lactation demands increase. From this point onward, adequate forage supply and improvements in pasture quality may have supported individual performance and mitigated the impacts of negative energy balance, allowing for a partial recovery of nutritional status by the end of the evaluation. The variation in the metabolic profile over time reflects the homeorhetic adjustments required to support late fetal growth, parturition, and the transition from gestation to lactation [8,27,30]. Additionally, it complements the assessment of productive performance, enabling the evaluation of the animals’ nutritional status under different levels of restriction [27,31]. Overall, the metabolites analyzed in our study confirm a more pronounced negative energy balance up to d30, evidenced by its reduction at this point with the resumption of glucose and serum protein levels, as well as a decrease in βHB and NEFA levels.

From d30 and d45 onwards, a partial recovery in IGF-1 and total cholesterol levels was observed, respectively. Higher levels of these metabolites may indirectly contribute to the early resumption of reproductive function by stimulating gonadotropic hormone secretion and follicular growth [6,32,33]. Additionally, the stabilization of BW from d30 onwards, along with reductions in BHB and NEFA levels, suggests an improvement in the negative energy balance. This enhancement in energy status can be attributed to increased nutrient availability and the resumption of feed intake capacity, leading to the decreased mobilization of body reserves due to the heightened demands of lactation. Consequently, it is likely that a greater proportion of energy was allocated to reproductive processes, favoring the resumption of ovarian activity and follicular growth. This effect was reflected in the DFPO observed in our study (average of 16.2 mm), which exceeded the threshold considered ideal for achieving high conception rates [34]. Larger follicles indicate a favorable nutritional status, with a greater availability of substrates for protein and energy metabolism, thereby promoting reproductive success [34,35].

Regarding the variables measured in the calves, our study indicates that maternal reserve mobilization was sufficient to ensure proper development during the final third of gestation, as reflected in birth weight and *Longissimus dorsi* fiber area, which were similar among calves born to cows receiving different treatments. Additionally, maternal milk production was similar across treatments, which did not contribute to differences in calf performance up to 60 days of age. The adequate levels of proteins and energy metabolites observed in the calves on d1 and d15 suggest efficient colostrogenesis and proper nutritional status [27,36]. Combined with maternal milk production, these factors contributed to optimized weight gain up to 60 days of age, reaching approximately 800 g/day.

## 5. Conclusions

The supplementation of beef cows during the last third of gestation, when grazing low-quality pastures, helps to alleviate the effects of negative energy balance on metabolic indicators. In this context, the inclusion of up to 42% dried distillers grains in the supplements is effective in mitigating the nutritional deficit and promoting better peripartum weight variation.

## Figures and Tables

**Table 1:** Ingredients and nutritional composition of supplements for pregnant Nellore cows in late gestation, supplemented with different levels of inclusion of dried distillers grains.

Item	Supplement			
	Control	0% DDG	42% DDG	84% DDG
	g/kg—as feed basis			
Corn meal	-	477	238	-
Soybean meal	-	363	182	-
DDG	-	-	420	840
Urea AS	-	80	80	80
Mineral mixture ^1^	1000	80	80	80
	g/kg—dry matter			
DM ^2^	-	855	820	819
OM ^2^	-	908	903	907
CP ^2^	-	437	437	429
apNDF ^2^	-	113	257	413
iNDF ^2^	-	15	32	62
RDP ^3^	-	342	314	286
RUP ^3^	-	72	108	144

^1^ Mineral mixture composition: Dicalcium phosphate 300 g/kg, Sodium chloride 253 g/kg, Sulfur flower 33.3 mg/kg, Magnesium oxide 16.7 mg/kg, Zinc sulfate 15.0 mg/kg, Copper sulfate 7.0 mg/kg, Manganese sulfate 5.0 mg/kg, Cobalt sulfate 0.5 mg/kg, Potassium iodide 5.0 mg/kg, Sodium selenite 0.1 mg/kg; ^2^ DM, dry matter; OM, organic matter; CP, crude protein; apNDF, neutral detergent fiber corrected for ash and protein; iNDF, indigestible neutral detergent fiber; ^3^ RDP and RUP, rumen degradable protein and rumen undegradable protein.

**Table 2:** Nutritional composition of *Urochloa decumbens* and forage availability for pregnant Nellore cows in late gestation, supplemented with different levels of inclusion of dried distillers grains.

Item ^1^	Month					
	June, d-90	July, d-60	August, d-30	September, d0	October, d30	November, d60
	g/kg—dry matter					
DM	494	620	641	579	258	268
OM	921	926	931	925	911	906
CP	38	35	33	54	102	87
apNDF	729	757	752	733	620	667
iNDF	295	332	310	341	270	239
	g/kg—body weight					
fDM	85.2	78.0	62.9	60.4	66.9	75.5
pdDM	58.1	49.6	38.1	37.7	42.0	50.0

^1^ DM, dry matter; OM, organic matter; CP, crude protein; apNDF, neutral detergent fiber corrected for ash and protein; iNDF, indigestible neutral detergent fiber; fDM, forage dry matter offered; pdDM, potentially digestible forage dry matter offered.

**Table 3:** Relative variation in body weight, body condition score, ribeye area, and rump fat thickness measured during peripartum period of Nellore cows supplemented or not with different levels of dried distillers grains during last trimester of gestation.

Item ^1^	Treatment ^2^				SEM	*p*-Value ^3^				
	Control	0% DDG	42% DDG	84% DDG		SUP	L	Q	Prd	Trt × Prd
Body weight, %					1.103	0.29	0.65	0.047	<0.001	0.07
Prepartum	3.80 ^b^	6.33 ^ab^	10.20 ^a^	5.76 ^b^						
Postpartum	−4.10 ^ab^	−7.32 ^b^	−3.04 ^a^	−4.67 ^ab^						
Body condition score, %					1.518	0.43	0.52	0.63	<0.001	0.68
Prepartum	−0.20	1.06	4.96	1.87						
Postpartum	−10.48	−10.8	−10.98	−9.78						
Ribeye area, %					3.663	0.65	0.72	0.23	<0.001	0.74
Prepartum	2.92	1.61	3.81	−0.14						
Postpartum	−15.90	−22.50	−13.55	−17.96						
Rump fat thickness, %					8.063	0.67	0.65	0.20	<0.001	0.41
Prepartum	2.49	11.22	19.38	14.75						
Postpartum	−50.98	−65.13	−45.91	−60.12						

^1^ Prepartum variation was calculated as the difference between calving (d-1) and initial (d-90) measurements, relative to the initial (d-90) measurement. Postpartum variation was calculated as the difference between final (d60) and calving (d-1) measurements, relative to the calving (d1) measurement. ^2^ The supplements were formulated to contain 40% of CP and were offered daily at a rate of 1 kg/animal for 85 days prior to calving. ^3^ The effects of supplementation (SUP), the linear inclusion (L) and quadratic inclusion (Q) of DDG levels, measurement period (Prd), and the interaction between treatment and measurement period (Trt × Prd). ^a, b^ Means with different superscripts within a row differ from each other at *p* < 0.05.

**Table 4:** Average concentration of serum urea nitrogen, total cholesterol, β-hydroxybutyrate, and non-esterified fatty acids measured during peripartum period in Nellore cows supplemented or not with different levels of dried distillers grains during last trimester of gestation.

Trt ^1^	Days Relative to Calving							SEM	*p*-Value ^2^				
	d-30	d-15	d1	d15	d30	d45	d60		SUP	L	Q	Day	Trt × Day
	Serum urea nitrogen, mg/dL							0.85	0.020	0.005	0.77	<0.001	<0.001
Control	9.1 ^B^	8.3 ^C^	11.0	8.3 ^BC^	11.5	12.4 ^AB^	9.1						
0% DDG	16.6 ^A^	18.8 ^A^	12.5	9.6 ^AB^	10.1	14.5 ^A^	7.5						
42% DDG	13.3 ^AB^	13.1 ^B^	11.8	12.2 ^A^	11.9	9.7 ^B C^	8.1						
84% DDG	12.2 ^AB^	12.5 ^B^	13.2	6.6 ^C^	11.0	8.9 ^C^	8.7						
*Overall ^3^*	*27.4 ^ab^*	*28.2 ^a^*	*26.0 ^ab^*	*19.7 ^c^*	*23.8 ^b^*	*24.3 ^b^*	*17.9 ^c^*						
	Total cholesterol, mg/dL							8.54	0.69	0.50	0.99	<0.001	0.036
Control	144.6	130.7	102.6 ^AB^	119.4	121.9 ^A^	131	121.2						
0% DDG	149.5	146.7	96.1 ^B^	106.9	111.8 ^AB^	136.2	124.5						
42% DDG	138.3	131.2	117.9 ^AB^	106.3	104.6 ^AB^	118.4	131.0						
84% DDG	134.6	132.4	123.0 ^A^	100.2	87.6 ^B^	127.7	119.1						
*Overall ^3^*	*141.7 ^a^*	*135.2 ^b^*	*109.9 ^d^*	*108.2^d^*	*106.5 ^d^*	*128.3 ^bc^*	*123.7 ^c^*						
	β-hydroxybutyrate, mmol/L							0.0504	0.62	0.87	0.25	0.004	0.033
Control	0.546 ^AB^	0.731 ^B^	0.511	0.499	0.479	0.478	0.422						
0% DDG	0.475 ^AB^	0.543 ^AB^	0.567	0.490	0.546	0.500	0.487						
42% DDG	0.427 ^A^	0.436 ^A^	0.440	0.522	0.422	0.451	0.445						
84% DDG	0.627 ^B^	0.601 ^AB^	0.554	0.563	0.400	0.513	0.423						
*Overall ^3^*	*0.519 ^ab^*	*0.578 ^a^*	*0.518 ^ab^*	*0.517 ^ab^*	*0.462 ^bc^*	*0.486 ^bc^*	*0.444 ^c^*						
	Non-esterified fatty acids, mmol/L							0.0482	0.049	0.84	0.39	<0.001	0.012
Control	0.473 ^B^	0.606 ^B^	1.393 ^B^	0.907	0.420	0.157	0.230						
0% DDG	0.139 ^A^	0.350 ^A^	1.096 ^AB^	0.866	0.446	0.25	0.119						
42% DDG	0.149 ^A^	0.236 ^A^	1.046 ^AB^	0.846	0.243	0.123	0.095						
84% DDG	0.402 ^B^	0.413 ^AB^	0.875 ^A^	0.872	0.263	0.169	0.154						
*Overall ^3^*	*0.291 ^d^*	*0.401 ^c^*	*1.103 ^a^*	*0.873 ^b^*	*0.343 ^cd^*	*0.175 ^e^*	*0.152 ^e^*						

^1^ The supplements were formulated to contain 40% of CP and were offered daily at a rate of 1 kg/animal for 85 days prior to calving. ^2^ The effects of supplementation (SUP), the linear inclusion (L) and quadratic inclusion (Q) of DDG levels, measurement period (day), and the interaction between treatment and measurement period (Trt × Day). ^3^ The overall means of repeated measures over time are presented in italics to distinguish them from the means of each treatment at each time point evaluated.. ^A, B, C^ Means followed by different uppercase superscript letters in the same column differ from each other at *p* < 0.05. ^a, b, c, d, e^ Means with different lowercase superscripts letters within a row differ from each other at *p* < 0.05.

**Table 5:** Milk yield of Nellore cows supplemented or not with different levels of dried distillers grains during late gestation and birth weight, metabolic profile, and muscle fiber area of their calves.

Item	Treatment ^1^				SEM	*p*-Value ^2^				
	Control	0% DDG	42% DDG	84% DDG		SUP	L	Q	Day	Trt × Day
Body weight, kg					2.36	0.35	0.16	0.97	<0.001	0.21
d1	37.6	35.9	36.6	34.9						
d60	86.6	87.8	81.3	77.5						
Total protein, g/dL					0.23	0.61	0.20	0.64	<0.001	0.21
d1	6.5	6.6	5.7	6.2						
d15	5.5	6.0	5.5	4.7						
Albumin, g/dL					0.02	0.42	0.14	0.73	0.004	0.85
d1	2.2	2.4	2.3	2.1						
d15	2.6	2.6	2.4	2.2						
Globulin, d/dL					0.19	0.80	0.28	0.72	<0.001	0.17
d1	4.3	4.4	3.5	4.0						
d15	2.9	3.3	3.3	2.5						
Glucose, mg/dL					5.03	0.85	0.39	0.71	0.38	0.60
d1	117.9	120.7	128.4	109.1						
d15	112.7	121.9	111.9	111.4						
IGF-1, ng/dL	230.6	244.4	247.9	282.0	19.64	0.32	0.26	0.61	-	-
Muscle fiber, μm^2^	703	671	735	612	62.5	0.70	0.50	0.30	-	-
Dams’ milk yield, kg	6.6	7.6	7.8	7.6	0.72	0.33	0.97	0.87	-	-

^1^ The supplements were formulated to contain 40% of CP and were offered daily to the calves’ dams at a rate of 1 kg/animal for 85 days prior to calving. ^2^ The effects of supplementation (SUP), the linear inclusion (L) and quadratic inclusion (Q) of DDG levels, measurement period (day), and the interaction between treatment and measurement period (Trt × Day). Sex and the interactions with treatment and day were not significant (*p* > 0.05).

## Data Availability

The data presented in this study are available upon request from the corresponding author.

## Abbreviations

DDG	Dried distillers grains
BW	Body weight
BCS	Body condition score
βHB	β-hydroxybutyrate
NEFAs	Non-esterified fatty acids
SUN	Serum urea nitrogen
RUP	Rumen undegradable protein
RDP	Rumen degradable protein
CP	Crude protein
DM	Dry matter
apNDF	Neutral detergent fiber corrected by ash and protein
iNDF	Indigestible neutral detergent fiber
pdDM	Potential digestible dry matter
fDM	Forage dry matter
